# Cholecystocutaneous fistula incidence, Etiology, Clinical Manifestations, Diagnosis and treatment. A literature review

**DOI:** 10.1016/j.amsu.2020.09.035

**Published:** 2020-10-02

**Authors:** Muhamad Zakaria Brimo Alsaman, Muhammad Mazketly, Mohammad Ziadeh, Owais Aleter, Ahmad Ghazal

**Affiliations:** a Faculty of Medicine, University of Aleppo, Aleppo, Syria; b Department of Radiology, Aleppo University Hospital, Aleppo, Syria; c Department of Surgery, Aleppo University Hospital, Syria

**Keywords:** Hepatobiliary disease, Gallstones and cholestasis

## Abstract

Cholecystocutaneous Fistula (CCF) is a type of external biliary fistula, which connects the gallbladder with the skin. Thilesus first described this phenomenon in 1670. There is usually a history of calculi in the gallbladder or neglected gallbladder disease.

The incidence of CCF is rare, most patients are elderly females with the mean age of 72.8 years old. They usually present with chronic calculus cholecystitis or a history of a previous surgical intervention.

US, CT, MRI, MRCP and (CT or X-ray) fistulogram are used to confirm the diagnosis. CT was more significant than US in identifying the track of the fistula and the fluid that runs throw it.

CCF patients presented with systemic symptoms (fever, nausea and vomiting) or local symptoms. RUQ region is the most common site of external opening.

Open cholecystectomy with excision of the fistulous tract is considered an acceptable option for treatment and it is curative in most cases.

However, laparoscopic approach can be another option with experience surgeons.

## Highlights

• Cholecystocutaneous Fistula (CCF) is a type of external biliary fistula, which connects the gallbladder with the skin.
• Most patients present with chronic calculus cholecystitis or with a history of a previous surgical intervention.
• Patients treated with conservative or surgical way.
• We clarified, and in numbers, the possible pathogens that might be the main cause of the CCF.
• A new comprehensive illustration of the CCF.

## 1 Introduction

Fistula is an abnormal condition, which results from abnormal connection between two epithelialized surfaces. Biliary fistulas are rare complications of gallstone, that connect between the biliary tract and other organs, there are two main groups of biliary fistulas: external and internal [1].

Internal biliary fistula connects the gallbladder with gastrointestinal tract, it is induced by chronic cholecystitis [2].

External biliary fistula connects the gallbladder with abdominal wall, it could be spontaneous, postoperative or post-traumatic or caused by iatrogenic injury of biliary tract [[1], [3]].

**Fig. 1:**
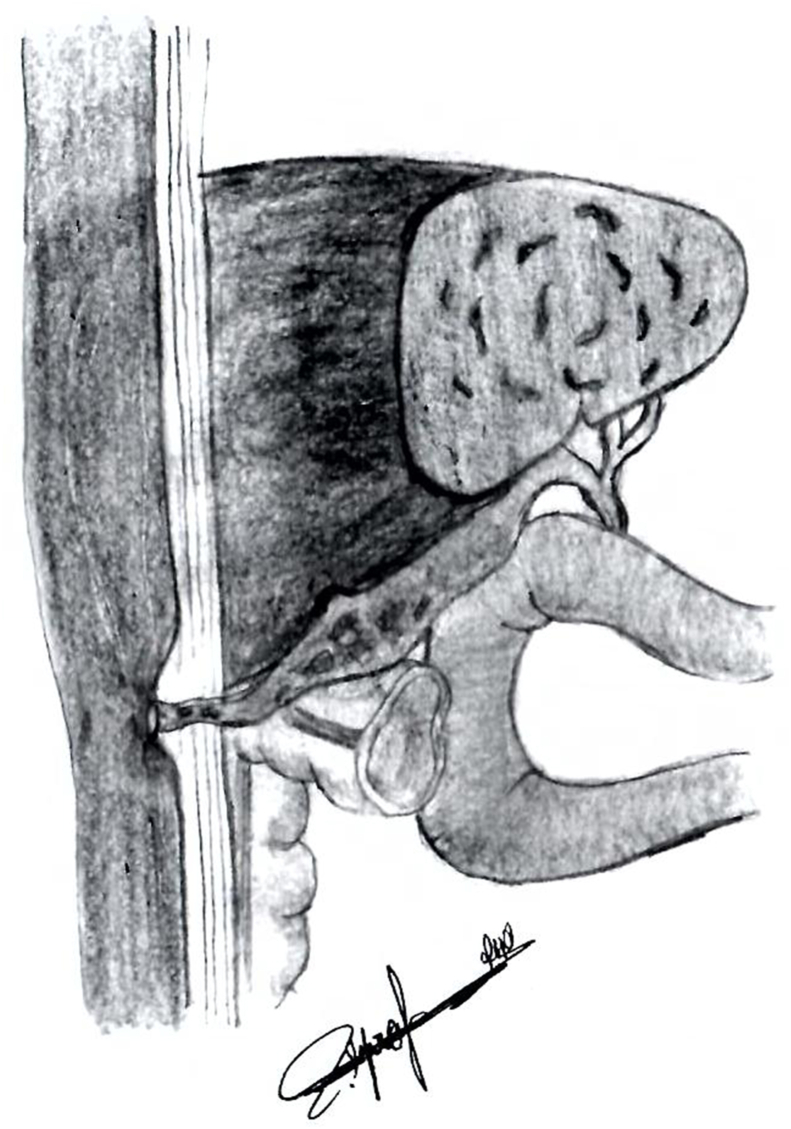
This figure is an illustration of Cholecystocutaneous fistula. It shows the fistula that connects the gallbladder with the skin.

Cholecystocutaneous fistula is a type of external biliary fistula, which connects the gallbladder with skin (Fig. 1).

Untreated or neglected gallbladder disease may lead to CCF are the main risk for CCF cases. Most patient presented with a history of calculi in the gallbladder or neglected gallbladder disease and they treated with conservative or surgical way.

There are less than 100 cases of cholecystocutaneous fistula reported in the medical literature [4]. The first reported case of CCF was in 1670 by Thilesus, who described this phenomenon for the first time.

CCF can be spontaneous after cholethiasis and neglected gallbladder disease (5), or following previous surgery such as percutaneous cholecystostomy drain removal [4].

This study reviews the Incidence, Etiology, Clinical Manifestations, Diagnosis and Management for Cholecystocutaneous Fistula patients, using data of pervious case report in the medical literature.

## 2 Methods

We conducted a literature search for relevant studies that have been published between 1954 and 2020 in Pubmed database by using the following search term “Cholecystocutaneous Fistula”. There were no restrictions on country, or language. Articles were excluded if they were animal studies or uncompleted data. We reviewed 48 case report articles that were published between 1954 and 2020.

### 2.1 Incidence and etiology

The incidence of CCF is rare, most patients are elderly females (M/F: 18/29) with the mean age of 72.8 years old (Table .1) (see Table 2).

**Table 1:** Age, sex, etiology, presentations, diagnosis and management of Cholecystocutaneous Fistula.

Authors	Age	Sex	Etiology	Presentations	Confirmed diagnosis	Management
Wang et al. [18]	86	M	Calculous cholecystitis	Pain and swelling in the RUQ	MRI	Open cholecystectomy
Rinzivillo et al. [5]	76	M	Calculous cholecystitis	tumefaction of the right hypochondrium,	MRI	Open cholecystectomy
El Tinay et al. [23]	78	M	Calculous cholecystitis	Fever, malaise and a right subcostal mass	CT	Open cholecystectomy
Ioannidis et al. [8]	71	M	Calculous cholecystitis	persistent bilious drainage from an old surgical scare, from surgical drainage, of the right upper abdominal quadrant	X-Ray fistulogram	Open cholecystectomy
Cheng et al. [32]	21	F	Calculous cholecystitis	Soft tissue defect at posterior trunk and sacral area after a major trauma	CT fistulogram	PTGBD
Chatterjee et al. [11]	45	F	Calculous cholecystitis	acute onset of pain and swelling in the right hypochondrium	X-Ray fistulogram	Open cholecystectomy
Ijaz et al. [10]	74	F	Calculous Cholecystitis	fevers, malaise and a right subcostal mass	CT	Open cholecystectomy
Ayoub et al. [26]	65	M	Gallstones	Swelling in the right hypochondriac area.	During the surgery	Open cholecystectomy
Pol et al. [21]	70	F	Gallstones	Discharging sinus in the right hypochondriac region	CT fistulogram	Laparoscopic cholecystectomy
Kassi et al. [6]	46	_	Gallstones	Painful, fluctuating, epigastric swelling of 15 days' duration.	CT	Open cholecystectomy
Bermúdeza et al. [30]	30	F	Gallstones	pain in the right hypochondrium of years evolution	X-Ray Fistulogram	Open cholecystectomy
Dixon et al. [28]	94	F	Gallstones	wound discharge and non-healing wound	CT	conservative management
Polite et al. [29]	70	F	Gallstones	acute on chronic midepigastric abdominal pain, associated with nausea and vomiting	Hepatobiliary iminodiacetic acid	Open cholecystectomy
Ozdemir et al. [24]	89	F	Gallstones	right upper abdominal pain and icterus	CT	Open cholecystectomy
Gordon et al. [33]	83	F	Gallstones	mild, intermittent right upper quadrant pain	CT	Open cholecystectomy
Khan et al. [34]	76	M	Gallstones	necrotizing fasciitis of anterior abdominal wall and cholecystocutaneous fistula	X-Ray fistulogram	Open cholecystectomy
Pezzilli et al. [17]	90	F	Gallstones	diarrhea and low-grade fever	CT	conservative management and Ct drainage of the purulent collection was also carried out
Aguilar et al. [35]	83	M	Gallstones	pain in the right upper quadrant and the appearance of a mass	CT	conservative management
Hawari et al. [9]	84	M	Gallstones	intermittent right upper quadrant abdominal pain, nausea, darkening of his urine, and increasing jaundice	X-Ray fistulogram	Open cholecystectomy
Sayed et al. [31]	85	F	Gallstones	soft and non-tender mass in her right flank.	MRCP	ERCP and sphincterotomy
Yüceyar et al. [36]	70	F	Gallstones	abscess formation in the right upper quadrant	_	Open cholecystectomy
Carragher et al. [37]	67	F	Gallstones	Right hypochondrial pain	CT	Conservative management
Hoffman et al. [38]	70	F	Gallstones	chronic epigastric pain.	During the surgery	Open cholecystectomy and choledocholithotomy
	73	F	Gallstones	ten-day history of dull epigastric pain and anorexia	During the surgery	Laparotomy
Jeffery et al. [39]	_	M	Gallstones	enlarging mass on the right upper abdomen	During the surgery	Open cholecystostomy
Schippers et al. [40]	75	M	Cholecystitis	_	_	_
Gerrard et al. [22]	80	F	Cholecystitis	acute cholecystitis	CT	Percutaneous cholecystectomy
Mughal et al. [19]	74	F	Cholecystitis	unremitting pain in the right shoulder that had progressed to the right side of the abdomen	During the surgery	Open cholecystectomy
				pain and swelling in the RUQ, and development of a discharging sinus within it	US + CT	Open cholecystectomy
Maynard et al. [7]	68	F	Cholecystitis	painful swelling in the right upper anterior abdominal wall.	CT	Open cholecystectomy
Jayasinghe et al. [41]	87	F	Cholecystitis	sepsis following a fall	CT	Open abscess drainage
Mughal et al. [19]	74	F	Cholecystitis	sepsis following a fall	CT	Open abscess drainage
Kapoor et al. [15]	45	M	Cholecystitis	right hypochondrial tenderness and bilious discharge from the scar	X-Ray fistulogram	Open Cholecystectomy
	65	M	Cholecystitis	discharging sinus from the anterior abdominal wall	CT	cholecystocutaneous fistula excision
Kim et al. [42]	72	F	Cholecystitis	persistent small volume discharge from the drain site	sinogram	cholecystocutaneous fistula excision
Flora et al. [27]	67	M	Cholecystitis	persisting discharge from what was thought to be an ‘abscess’ in the right hypochondrium	CT	Open cholecystectomy
Malik et al. [43]	76	F	Cholecystitis	acute cholecystitis	CT	laparoscopic cholecystectomy
Cruz et al. [25]	81	M	Cholecystitis	right upper abdominal pain	During the surgery	Open cholecystectomy
Khan et al. [44]	90	F	Cholecystitis	RUQ swelling	CT	Open cholecystectomy
Dutriaux et al. [45]	65	M	Cholecystitis	inflammatory and ulcerated lesion located on his right flank	Systemic Pathology Exam	Open cholecystectomy
Vasanth et al. [46]	_	_	Cholecystitis	_	_	_
Mathonnet et al. [47]	87	F	Cholecystitis	right hypochondrial pain.	X-Ray fistulogram	Open cholecystectomy
Sedgwick et al. [48]	76	M	Cholecystitis	acute cholecystitis	upper abdominal pain	ERCP
Pripotnev et al. [16]	85	F	Cholecystocutaneous fistula developing after the removal of a percutaneous drain for the treatment of acute cholecystitis	sharp intermittent epigastric and right upper quadrant pain radiating to the central back.	During the surgery	laparoscopic cholecystectomy
Sodhi et al. [13]	66	F	Adenocarcinoma of gallbladder	Pain in the right hypochondrium.	X-Ray fistulogram	Conservative management (chemotherapy)
Serrano et al. [49]	83	M	Papillary adenocarcinoma	abdominal pain in the right upper quadrant associated with the oozing of hematic purulent content through an orifice in the abdominal wall located in the right hypochondrium	CT	radical cholecystectomy
Andersen et al. [50]	89	F	–	abscess in the right breast	ERCP	Open cholecystectomy
Lofgren et al. [4]	60	F	Severe cholecystitis the year prior that was managed by a percutaneous cholecystostomy drain	Shortness of breath, RUQ pain, nausea, emesis, and a fever.	CT with oral contrast	Robotic cholecystectomy
Seoane et al. [51]	83	F	Spontaneous external biliary fistula and a history of ERCP three months before	abdominal pain and fever and mass in the RUQ	During the surgery	Open cholecystectomy
Murphy et al. [52]	80	M	Cholecystitis	swelling on the anterior abdominal wall in the right upper quadrant extending over the right costal margin	During the surgery	subtotal cholecystectomy
Grimes et al. [14]	70	F	traumatic rupture of the gallbladder	pain in the upper abdomen	X-Ray	cholecystocutaneous fistula excision
**Mean age**	72.8					
**Standard Deviation**	15

**Table 2:** Presenting microorganism found in cholecystocutaneous fistula cases.

Authors	Microorganism
Lofgren et al. [4], Micu et al. [12], El Tinay et al. [23] Jayasinghe et al. [41] Fabbi et al. [53], Ioannidis et al. [8], Hoffman et al. [38], Orville et al. [14]	*Escherichia coli*
Flora et al.(27), Murphy et al. [52], Ijaz (10)	Coliforms
El Tinay et al. [23], Ioannidis et al. [8]	Klebsiella pneumonia
Kassi et al. [6]	*Helicobacter pylori*
Ioannidis et al. [8]	Staphylococcus hominis,
Cheng et al. [32]	*Staphylococcus aureus*
Hawari (9)	Strepto-coccus milleri
Micu et al. [12]	*Enterococcus faecalis*
Lofgren et al. [4]	Bacteroides fragilis
Mathonnet et al. [47]	Fragils
Hoffman et al. [38]	Proteus mirubilis
Orville et al. [14]	Viridans streptococci (enterococci)

CCF mainly is a result of neglected gallbladder disease.

Most cases presented with chronic calculus cholecystitis or with a history of previous surgical intervention as a case of subtotal cholecystectomy for acute cholecystitis [[6], [7], [8], [9], [10], [11]].

Increasing pressure in the gallbladder after calculus cholecystitis, which leads to fistula formation, is believed to be the pathophysiology mechanism of this condition.

Most common cases of CCF are related to bacterial infection in the gallbladder, but there are few cases of CCF arising from adenocarcinoma of gallbladder [[12], [13]].

*Escherichia coli* is the most common microorganism found in cholecystocutaneous fistula cases followed by Coliforms and klebsiella pneumonia. (Table. 2).

Also retained stones after laparoscopic cholecystectomy and traumatic rupture of the gallbladder are considered as predisposing factors for cholecystocutaneous fistula [[14], [15]].

### 2.2 Clinical Manifestations

The general condition of the patient variable is depending on the age and past medical history. There is usually a history of calculi in the gallbladder or neglected gallbladder disease.

CCF patients presented with systemic symptoms or specific symptoms.

The patients showed systemic symptoms such as: fever, nausea and vomiting [[12], [16], [17]].

However, patients may present with pain and swelling in the RUQ or epigastric region [[6], [18]], discharging sinus from the anterior abdominal wall [[15], [19]], erythematous mass [20], subcostal abscess [18] (Table .1). The most common site of external opening is in the right upper quadrant of the abdominal wall. It can also be seen in right flank, right subcostal area, epigastric region, right breast, para-umbilical site (Table .3).

**Table 3:** Presenting site of external opening.

Location	Authors
RUQ	Ayoub et al. [26], Lofgren et al. [4], Seoane et al. [51], pol et al. [21], Wang et al. [18], Rinzivillo et al. [5], Gerrard et al. [22], El Tinay et al. [23], Mughal et al. [19], Maynard et al. [7], Bermúdez et al. [30], Pripotneva et al. [16], Kapoor et al. [15], Polite et al. [29], Kim et al. [42], Sodhi et al. [13], Ioannidis et al. [8], Cheng et al. [32], Serrano et al. [49], Gordon et al. [33], Khan et al. [34], Pezzillia et al. [17], Hawari et al. [9], Chatterjee et al. [11], Flora et al. [27], Ijaz et al. [10], Malik et al. [43], Yüceyar et al. [36], Carragher et al. [37], Sedgwick et al. [49], Abril et al. [54], Jeffrey et al. [39], Orville et al. [14],
Right flank	Sayed et al. [31], Khan et al. [44], Jayasinghe et al. [41]
Right subcostal area	Ozdemir et al. [24], Cruz et al. [25], Hoffman et al. [38]
Epigastric region	Micu et al. [12], Kassi et al. [6]
Right breast	Andersen et al. [50]
para-umbilical	Dixon et al. [28]

### 2.3 Diagnosis

CCF is diagnosed usually using imaging studies, or exploratory laparotomy in special cases.

### 2.4 Imaging studies include

• Ultrasonography (US)
• Computed tomography (CT)
• Fistulogram
• Magnetic resonance imaging (MRI)

US provides good assessment for CCF diagnosis by showing abnormal findings such as abscess, gallbladder stones, edema, thickened in gallbladder wall and dilated biliary ducts [[5], [15], [17], [21]] but often fails to confirm the CCF diagnosis. In a few cases, US was able to demonstrate the track of CCF [[12], [19]].

CT confirmed the diagnosis by identifying the track between the gallbladder and the skin in several cases [[22], [23], [24], [25]]. Furthermore, CT fistulogram can also show the track of CCF which confirm the diagnosis [21]. CT couldn't identify the track of CCF in a few cases where it just showed abnormal findings which point toward the diagnosis [[19], [23], [26], [27]].

In total, CT was more significant than US in identifying the track of the fistula and the fluid that runs throw it.

MRI was able to detect gallstones, gallbladder perforation, and the fluid, which extruded through the abdominal wall [[5], [18]].

MRI could be more accurate when CT detects no abnormalities.

MRCP increases the confirmed cases [[7], [17], [19], [28]]. The results of MRCP are identical to US and CT.

Hepatobiliary iminodiacetic acid scan was used in two cases; it failed to demonstrate the fistula in one case (4) and showed obstruction in biliary tracks in the second one [29].

In addition, exploratory laparotomy may be the only diagnosis methods especially in old reported cases, in poor countries or with shortage of appropriate equipment and laparoscopic experience.

CT Fistulogram or X-ray Fistulogram have been used to demonstrate track of the fistula accurately [[14], [21], [30]], although in most cases Fistulogram was not used and the diagnose was made by another method.

There are problem and the difficulties facing the diagnosis such as absence of radiological expertise, it is uncommon to consider CCF as deferential diagnosis since it is a rare condition and presence of mucous discharge rather than yellowish discharge if there is an obstruction in the cystic duct makes it hard to diagnose especially in the early stages.

### 2.5 Management

The management of CCF vary according to disease severity, age, and the patient's preference.

There is no standard base line management for cholecystocutaneous fistula, due to the fewness of the number of cases and the differences in patients’ illness quality.

The medical literature mentioned different ways of CCF management, either conservative or surgical management.

### 2.6 Conservative management

Conservative management includes antibiotics, fluids or ERCP.

Percutaneous abscess drainage is performed immediately with the guidance of CT, or US, then all patients should receive management by antibiotics to manage infections and cholecystitis [[4], [7], [16], [17], [23], [26]].

Conservative management is performed to elderly patients who are unable to tolerate surgery [17].

Few cases were treated by using endoscopic retrograde cholangiopancreatography (ERCP) for CCF treatment, by removing calculi using ERCP balloon trawl and sphincterotomy [31].

Chemotherapy is applied for CCF patients due to carcinoma of the gallbladder [13].

Percutaneous transhepatic gallbladder drainage (PTGBD) can be applied to treat CCF. Where the fistula heals under secondary intention after removing drainage tube [32].

Conservative management cured few cases, helped relieve symptoms and improved patient's condition.

Surgical management:

Surgical management includes open cholecystectomy and laparoscopic cholecystectomy.

Open cholecystectomy with excision of the fistulous tract is considered as a standard option for management and it is curative in most cases. In the other hand, laparoscopic cholecystectomy with excision of the tract can be another acceptable and preferable option with advance experienced laparoscopic surgeons.

Presence of comorbidity in patients lead to failure of healing, also difficulty in performing surgery for cachectic or elderly patients.

Lack of adequate surgical experience can lead to serious complications and difficulty in treatment.

## 3 Conclusion

CCF diagnosis and management represent one of the surgical obstacles, which we still encountered from time to time.

We noticed through our review different ways of diagnosis and management. Most of them were dependent on the surgical experience and the advanced medical investigation equipment.

In conclusion, there is no standard ways for diagnosis and management of CCF but according to our review; we think that each surgeon should choose the best way to deal with CCF patients depending on patients ‘quality, available equipment and advanced experienced surgeons.

## Ethical approval

Not required.

## Sources of funding

The authors have not declared a specific grant for this research from any funding agency in the public, commercial or not-for-profit sectors.

## Author contribution

MZBA, MM, MZ, OA: Writing - Original Draft. MM, MZBA: Writing - Review & Editing. MM: Formal analysis and Resources. MZBA: Validation and Visualization. AG: Supervision and Project administration. MZ: corresponding author.

## Guarantor

Mohammad Ziadeh.

## Funding

The authors have not declared a specific grant for this research from any funding agency in the public, commercial or not-for-profit sectors.

## Ethics

Not required.

## Patient consent for publication

Not required.

## Provenance and peer review

Not commissioned, externally peer reviewed.

## Declaration of competing interest

None declared.
